# Evaluation of Genetic Polymorphism of *Leishmania (V.) braziliensis* Isolates Obtained from the Same Patient before and after Therapeutic Failure or Reactivation of Cutaneous Lesions

**DOI:** 10.1155/2012/808132

**Published:** 2012-12-11

**Authors:** Cibele Baptista, Armando de Oliveira Schubach, Maria de Fatima Madeira, Luciana de Freitas Campos Miranda, Andressa Guimarães de Souza Pinto, Juliana Helena da Silva Barros, Fatima Conceição-Silva, Maria Ines Fernandes Pimentel, Raquel da Silva Pacheco

**Affiliations:** ^1^Laboratório de Vigilância em Leishmanioses, Instituto de Pesquisa Clínica Evandro Chagas, Fundação Oswaldo Cruz, Avenida Brasil 4365, 21040-900 Rio de Janeiro, RJ, Brazil; ^2^Laboratório de Imunoparasitologia, Instituto Oswaldo Cruz, Fundação Oswaldo Cruz, Avenida Brasil 4365, 21040-900 Rio de Janeiro, RJ, Brazil; ^3^Laboratório de Sistemática Bioquímica, Instituto Oswaldo Cruz, Fundação Oswaldo Cruz, Avenida Brasil 4365, 21040-900 Rio de Janeiro, RJ, Brazil

## Abstract

The aim of this study was to investigate genetic polymorphism in *Leishmania braziliensis* population previously typed through isoenzyme electrophoresis, isolated from the same patient in two different moments: (A) before the beginning of treatment and (B) after treatment failure to meglumine antimoniate or reactivation after successful initial treatment. Fifteen pairs of isolates were assessed using the polymorphic molecular marker LSSP-PCR and following the phenetic analysis. The genetic profiles of the 30 samples were grouped in four clusters. Only two patients presented total identity in the A and B isolates. Most isolates presented similarity coefficients varying from 0.63 to 0.91. In this group of patients genetic polymorphisms could be observed indicating low similarity between the pairs of isolates. The results demonstrate the existence of genetic polymorphism between the samples isolated before treatment and after reactivation or treatment failure, suggesting a possible differentiation of the structure of the original parasite population which could be involved in the mechanisms of resistance to treatment or reactivation of lesions in the ATL. This phenomenon is important, although other factors also could be involved in this context and are discussed in this paper.

In Brazil, American tegumentary leishmaniasis (ATL) is widespread and presents regional particularities, such as the occurrence of severe forms and resistance to treatment [1, 2]. There are few drugs available for ATL treatment. Pentavalent antimony is still the drug of first choice in the form of pentavalent antimonials [3] and in Brazil, it is used as meglumine antimoniate—Glucantime [4].

Response to the treatment of ATL is normally favorable but cases of therapeutic failure or clinical reactivation have been reported in some endemic areas. The therapeutic failure is defined by the absence of lesion epithelialization after treatment; otherwise, reactivation is characterized by the reappearance of papules on ATL cutaneous scars or around it which may happen months or years after initial clinical healing [4, 5]. The emergence of drug resistance becomes a major challenge in leishmaniasis [6, 7]. These challenges are enhanced with the occurrence of coinfection with HIV, increased migration, changes in the environment, difficulties in controlling the epidemics, introduction of new species, or emergence of subpopulations [8, 9].

Aiming at detecting genetic polymorphism among parasites from patients that presented therapeutic failure or reactivation of cutaneous lesions, the technique of low stringency single-specific primer-polymerase chain reaction (LSSP-PCR) was used in 15 pairs of isolates of *Leishmania braziliensis* separated in two groups: samples A—isolates obtained from initial lesions (before treatment with pentavalent antimony) and samples B—isolated from lesions of the same patients with treatment failure to pentavalent antimony or reactivation after successful initial treatment. 

The patients studied were from IPEC/FIOCRUZ and the study was approved by the Research Ethics Committee under number 0016.0.009-02. The *Leishmania* isolation and characterization by *Multilocus enzyme electrophoresis* (MLEE) following protocols were previously defined [10]. 

DNA was extracted from the samples according to protocols previously described [11, 12]. DNA was amplified using a pair of primers (BI: 5′-GGGGTTGGTGTAATATAGTGG-3′ and B2: 5′-CTAATTGTGCACGGGGAGG-3′), directed against the variable region of kDNA minicircles (mitochondrial DNA) of *Leishmania braziliensis* complex species (*Viannia *subgenus).

Fragments of 750 bp of kDNA of* Leishmania braziliensis* obtained by PCR were purified using Wizard PCR Preps system (Promega) following manufacturer's instructions. 

 LSSP-PCR reaction was carried out from the amplification of the 750 bp fragment with a single-specific primer using the sequence 5′-GGGGTTGGTGTAATATAGTGG-3′. Amplification was performed like previously described [12]. The amplification products were analyzed on 2% agarose gel or 1.8% agarose (Sigma) gel visualized with ethidium bromide under UV light. Reproducibility of LSSP-PCR method was assessed after three repetitions under identical conditions with retention of observed profiles.

LSSP-PCR bands varying from 200 to 750 bp were compared using the Simple Matching coefficient of similarity to determine the proportion of mismatched bands between pairs of isolates. The similarity matrix was transformed into a dendrogram using the UPGMA algorithm. Phenetic analysis was performed with the NTSYS-pc program (version 2.02, Exeter Software, Setauket, NY, USA). 

All patients presented ATL cutaneous form and the details were given in the Table 1. All isolates (samples A and B) were taxonomically classified as *Leishmania braziliensis* by MLEE. The samples analyzed by LSSP-PCR presented genetic polymorphism showing profiles with different degrees of complexity. 

Molecular biology techniques are useful tools for species identification and analysis of genetic diversity in *Leishmania* parasites. To our knowledge, this is the first work to report LSSP-PCR analysis in paired isolates from the same lesion of patients with ATL acquired before the beginning of treatment and after treatment failure or reactivation of the cutaneous lesion. By LSSP-PCR, genetic polymorphisms have been detected in samples from patients with typical and atypical clinical manifestations of ATL, including parasites from lesions after reactivation [13], demonstrating that LSSP-PCR is a sensitive technique for the investigation of intrapopulation genetic variability. Our research group using LSSP-PCR also confirmed intrapopulation genetic variability, distinguishing between two isolates from the same patient before and after reactivation [13]. 

After analysis of a total of 12 bands, the samples of this study were grouped into four clusters. Isolate 13 A shared 100% characters in common with the reference strain of *Leishmania braziliensis.* Intrapair genetic similarity (A and B) was 100% in pairs of isolates 2 and 3. The similarity of the others ranged from 0,63 to 0,91. The pairs of isolates genetically more differentiated were 5, 6, 12, and 14, whose A and B isolates were grouped in different clusters (Figure 1).

It is known that lesion reactivation can occur in 16% of cases under leishmaniasis treatment [14] and the persistence of parasites in blood and ATL scars also has been demonstrated [2, 15, 16]. History of previous or irregular treatment to leishmaniasis, presence of three or more lesions, long time of evolution of the disease, and also the presence of comorbidities may be associated to therapeutic failure [17]. However, other factors can be involved in the same cases of therapeutic failures without presence of these conditions. Recently similar and divergent genetic profiles were detected by LSSP-PCR in *L. braziliensis* isolated from HIV-infected and non-HIV-infected patients, demonstrating that the immune system also plays a role in the parasite population recovered from lesions [18]. 

In this study, all patients presented clinical manifestations of cutaneous leishmaniasis. Only two patients presented disseminated cutaneous lesions and their isolates (15 and 9) were grouped in clusters 2 and 3, respectively. All accompanied patients obtained clinical healing after retreatment. Five patients experienced clinical cure after final retreatment with second-choice drugs (amphotericin B or pentamidine) of whose three patients received initial treatment with intralesion injections. The other patients were cured with repetition of the first therapeutic schedule. Treatments using low doses or alternative schedules of antimony have been demonstrated successfully, especially in patients with comorbidities, children, or elderly people where high doses could be potentially more toxic [14]. 

The molecular marker used in the present study has been used in investigations of intrapopulation genetic variability as an important tool to demonstrate polyclonality and tropism of parasite populations [13–19].

In the present study 13.33% (4/30) of the samples showed similarity index equal to 1, which includes the pairs of isolates from patients 2 and 3 (A and B). Such pair of isolates was obtained from patients presenting therapeutic failure after 7 months (patient 3) and 14 months (patient 2). Two out of four patients that presented therapeutic failure were grouped in cluster 1 and the others in cluster 3. The time between the first and second isolations ranged from 18 to 21 months, suggesting that the elapsed time in different phases of clinical development may influence the genetic profiles detected. In the other samples a greater or lesser intrapair similarity could be detected with greater genetic diversity observed in isolates 4, 5, 6, 12, and 14. 

In Brazil, the therapeutic response to antimonials is considered favorable in most leishmaniasis cases, with variable healing rates. However, therapeutic failure in visceral and tegumentary leishmaniasis is a concern problem in many endemic areas [2, 17, 20]. From 1998 to 2010, cases of reactivation and treatment failure in patients diagnosed with ATL in the Evandro Chagas Clinical Research Institute-Oswaldo Cruz Foundation (IPEC-Fiocruz/Rio de Janeiro) have been notified in 15% of the patients. The mechanism involved in the poor response to therapy in leishmaniasis remains unclear to date and monitoring of these cases may help to understand the factors associated with drug resistance [21]. Different parasite subpopulations may be involved in the transmission cycle and the emergence of more resistant populations or able to cause atypical clinical manifestations should be detected in an attempt to control such eventual cases [22]. The four clusters assessed in this study grouped isolates from patients with reactivation after successful initial treatment. 

The differentiation in the structure of the original parasite population could be involved in the resistance mechanisms to treatment or lesions reactivation in the LTA, furthermore the phenomenon of the endogenous generation of new polymorphisms, particularly in kinetoplast DNA minicircles, which are considered to evolve more rapidly than nuclear DNA [23, 24]. The emergence of minor parasite subpopulations as a consequence of selection *in vivo *or propagation *in vitro* is difficult to ascertain in natural infections or recurrence of infectious and cannot be ruled out [9]. Using eight enzymatic loci no isoenzymatic variants were observed corroborating the fact that the LSSP-PCR is the molecular marker, highly polymorphic. Further molecular studies added to *in vitro* sensitivity phenotype to pentavalent antimony and immunological mechanisms of the host could be useful tools for a better understanding of these human cases of initial treatment failure or clinical recurrence of ATL. 

## Figures and Tables

**Figure 1:**
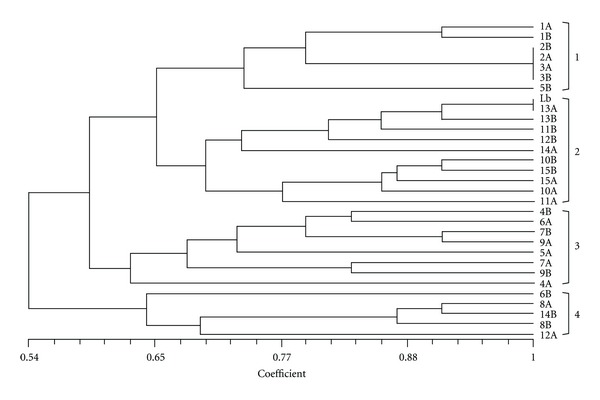
UPGMA dendrogram based on the LSSP-PCR profiles of samples isolated from lesion before treatment (A) and after reactivation (B). Lb: *L*. (*V*.) *braziliensis* reference strain.

**Table 1:** Data from 15 patients with ATL with failure to treatment or reactivation.

Patient and isolate number	Clinical form/*n*° of lesions	Time between first and second isolations (months)	Condition after first treatment	Drug used in the final retreatment
1 A	CL	6	Reactivation	Pentamidine
1 B	1			
2 A	CL	14	Treatment failure	Meglumine antimoniate
2 B	5			
3 A	CL	7	Treatment failure	Abandon
3 B	2			
4 A	CL	21	Treatment failure	Amphotericin B
4 B	1			
5 A	CL	10	Reactivation	Meglumine antimoniate
5 B	6			
6 A	CL	5	Reactivation	Meglumine antimoniate
6 B	2			
7 A	CL	18	Treatment failure	Anfotericina B
7 B	1			
8 A	LC	10	Reactivation	Meglumine antimoniate
8 B	2			
9 A	DCL	13	Reactivation	Anfotericina B
9 B	>ten lesions			
10 A	CL	13	Reactivation	Anfotericina B
10 B	1			
11 A	CL	6	Reactivation	Meglumine antimoniate
11 B	1			
12 A	CL	19	Reactivation	Meglumine antimoniate
12 B	2			
13 A	CL	10	Reactivation	Meglumine antimoniate
13 B	3			
14 A	CL	27	Reactivation	Meglumine antimoniate
14 B	1			
15 A	DCL	13	Reactivation	Meglumine antimoniate
15 B	>ten lesions			

CL (cutaneous leishmaniasis); DCL (disseminated cutaneous leishmaniasis).

Except patient 1 that received 10 mg Sbv/Kg/day on the first treatment, the others received low doses of pentavalent antimony (5 mg Sb/kg/day) with continuous or intermittent schedules or also intralesional injection (patients 4, 5, 6, 7, 10, and 12). All patients were retreated with repetition of the first therapeutic schedules. Five patients (1, 4, 7, 9, and 10) required one-third treatment with drugs of second line.

## References

[1] Marzochi MC, Marzochi KB (1994). Tegumentary and visceral leishmaniases in Brazil: emerging anthropozoonosis and possibilities for their control. *Cadernos de Saúde Pública*.

[2] De Oliveira Schubach A, Feldman Marzochi KB, Soares Moreira J (2005). Retrospective study of 151 patients with cutaneous leishmaniasis treated with meglumine antimoniate. *Revista da Sociedade Brasileira de Medicina Tropical*.

[3] Frézard F, Demicheli C, Ribeiro RR (2009). Pentavalent antimonials: new perspectives for old drugs. *Molecules*.

[4] Ministério da Saúde (Brasil) (2010). *Manual de Vigilância da Leishmaniose Tegumentar Americana, Secretaria de Vigilância em SaúDe*.

[5] Oliveira-Neto MP, Mattos M, De Souza CDSF, Fernandes O, Pirmez C (1998). Leishmaniasis recidiva cutis in New World cutaneous leishmaniasis. *International Journal of Dermatology*.

[6] Guerin PJ, Olliaro P, Sundar S (2002). Visceral leishmaniasis: current status of control, diagnosis, and treatment, and a proposed research and development agenda. *Lancet Infectious Diseases*.

[7] Sundar S, More DK, Singh MK (2000). Failure of pentavalent antimony in visceral leishmaniasis in India: report from the center of the Indian epidemic. *Clinical Infectious Diseases*.

[8] Sereno D, Cordeiro da Silva A, Mathieu-Daude F, Ouaissi A (2007). Advances and perspectives in *Leishmania* cell based drug-screening procedures. *Parasitology International*.

[9] Pacheco RS, Martinez JE, Valderrama L, Momen H, Saravia NG (1995). Genotypic polymorphisms in experimental metastatic dermal leishmaniasis. *Molecular and Biochemical Parasitology*.

[10] Cupolillo E, Grimaldi G, Momen H (1994). A general classification of new world *Leishmania* using numerical zymotaxonomy. *American Journal of Tropical Medicine and Hygiene*.

[11] Santos de Oliveira F, Valete-Rosalino CM, de Oliveira Schubach A, da Silva Pacheco R (2010). kDNA minicircle signatures of *Leishmania (Viannia) braziliensis* in oral and nasal mucosa from mucosal leishmaniasis patients. *Diagnostic Microbiology and Infectious Disease*.

[12] Ferreira GA, Soares FCS, Vasconcellos SA (2007). Discrimination of *Leishmania braziliensis* variants by kDNA signatures produced by LSSP-PCR. *Journal of Parasitology*.

[13] Baptista C, Schubach AO, Madeira MF (2009). *Leishmania (Viannia) braziliensis* genotypes identified in lesions of patients with atypical or typical manifestations of tegumentary leishmaniasis: evaluation by two molecular markers. *Experimental Parasitology*.

[14] Oliveira-Neto MP, Schubach A, Mattos M, Goncalves-Costa SC, Pirmez C (1997). A low-dose antimony treatment in 159 patients with American cutaneous leishmaniasis: extensive follow-up studies (up to 10 years). *American Journal of Tropical Medicine and Hygiene*.

[15] Vergel C, Palacios R, Cadena H (2006). Evidence for *Leishmania (Viannia)* parasites in the skin and blood of patients before and after treatment. *Journal of Infectious Diseases*.

[16] Schubach A, Marzochi MCA, Cuzzi-Maya T (1998). Cutaneous scars in American tegumentary leishmaniasis patients: a site of *Leishmania (Viannia) braziliensis* persistence and viability eleven years after antimonial therapy and clinical cure. *American Journal of Tropical Medicine and Hygiene*.

[17] Rodrigues AM, Hueb M, Rodrigues Dos Santos TAR, Fernandes Fontes CJ (2006). Factors associated with treatmet failure of cutaneous leishmaniasis with meglumine antimoniate. *Revista da Sociedade Brasileira de Medicina Tropical*.

[18] de Oliveira FS, Valete-Rosalino CM, de Oliveira Schubach A, de Fátima Madeira M, da Silva Pacheco R (2012). Genetic polymorphism in *Leishmania (Viannia) braziliensis* detected in mucosal leishmaniasis of HIV-infected and non-HIV-infected patients. *Transactions of the Royal Society of Tropical Medicine and Hygiene*.

[19] Alvarenga JSC, Ligeiro CM, Gontijo CMF (2012). KDNA genetic signatures obtained by LSSP-PCR analysis of *Leishmania (Leishmania) infantum* isolated from the New and the Old World. *PLoS ONE*.

[20] Unger A, O’Neal S, Machado PRL (2009). Association of treatment of American cutaneous leishmaniasis prior to ulcer development with high rate of failure in Northeastern Brazil. *American Journal of Tropical Medicine and Hygiene*.

[21] Ponte-Sucre A (2003). Physiological consequences of drug resistance in *Leishmania* and thier relevance for chemotherapy. *Kinetoplastid Biology and Disease*.

[22] Laurent T, Rijal S, Yardley V (2007). Epidemiological dynamics of antimonial resistance in *Leishmania donovani*: genotyping reveals a polyclonal population structure among naturally-resistant clinical isolates from Nepal. *Infection, Genetics and Evolution*.

[23] Brown WM, George M, Wilson AC (1979). Rapid evolution of animal mitochondrial DNA. *Proceedings of the National Academy of Sciences of the United States of America*.

[24] Simpson L (1987). The mitochondrial genome of kinetoplastid protozoa: genomic organization, transcription, replication, and evolution. *Annual Review of Microbiology*.

